# Group size and composition of brown howler monkeys (*Alouatta guariba*) in three fragments of Araucaria Pine Forest in southern Brazil

**DOI:** 10.1007/s10329-026-01283-y

**Published:** 2026-08-07

**Authors:** Amanda Letícia Borges, Gabriel Rangel Santos-Mariano, Pedro Augusto Andrich Mota, Samuel Barbarine da Silva, Mário Retondo, Lucas M. Aguiar

**Affiliations:** 1 Laboratório de Símios, Departamento de Zoologia, Universidade Federal do Paraná (UFPR), Coronel Francisco H. dos Santos Av., 100., Curitiba, 81531-980 Paraná Brazil; 2 Programa de Pós-graduação em Zoologia, Departamento de Zoologia, UFPR, Curitiba, Paraná Brazil; 3 Programa de Pós-graduação em Biodiversidade Neotropical, Universidade Federal da Integração Latino-Americana (UNILA), Foz do Iguaçu, Paraná Brazil

**Keywords:** *Alouatta guariba clamitans*, Ombrophilous Mixed Forest, Reproductive ratio, Social organization, Threatened species

## Abstract

Brown howler monkeys (*Alouatta guariba*) are increasingly threatened by habitat loss, sylvatic yellow fever, and negative human interactions. In southern Brazil, brown howlers still inhabit some fragments of the endangered Araucaria Pine Forest (APF), but basic information on their social organization remains scarce. We surveyed brown howler monkey groups to assess their group size and composition in three nearby similarly sized peri-urban APF fragments in Curitiba, southern Brazil. Using Generalized Linear Mixed Models, we investigated whether the number of males, females and immatures could be predicted by the number of individuals in each of these sex-age categories, along with group size. Monthly expeditions were conducted over 11–13 months, using multiple walks in each fragment to survey howler monkeys’ groups. We surveyed 17 groups over 332 km of fieldwork. Group sizes ranged from two to eight individuals (mean 4.88 ± 1.65 SD), sex and age proportion was 1 adult male: 0.5 subadult male: 1.7 adult females: 1.2 juvenile: 0.4 infant, and the immature-to-female ratio was 0.96. We found that group size was a significant positive predictor for the number of adult females, juveniles and immatures overall, suggesting a general increase in fitness in larger groups, but not for the number of males and infants. Our findings indicate the presence of a small established population of brown howler monkeys in relatively small APFs, with population parameters in the typical range of the species, but an immature-to-female ratio more similar to populations of stagnanting growth, pointing to the necessity to implement measures to conserve and connect this population in an endangered habitat.

## Introduction

Howler monkeys (*Alouatta* spp.) are large, arboreal, diurnal Platyrrhines with the widest geographic distribution among American primates, ranging from southern Mexico to northwestern Uruguay (Crockett and Eisenberg 1987; Jardim et al. 2019; Neville et al. 1988). As the most folivorous of the Platyrrhines, howlers have evolved life-history strategies shaped by their low-energy diet, relying on behavioral and physiological adaptations to conserve energy (Milton 1980).

Group competition in howlers and other markedly folivorous primates is primarily driven by sexual conflict, although competition for high-quality foods may also play a role (Chaves and Bicca-Marques 2013; Kamilar and Ledogar 2011; Knopff and Pavelka 2006; Snaith and Chapman 2007). Group size, age structure and sex ratio are key aspects of howler monkey social organization (Pollard and Blumstein 2008). Although group size can confer advantages in terms of group defense (Markham and Geskiere 2017), the number of females per group—often considered the core of primate social units —is a critical predictor of group size, given the central role of females in primate social evolution (Lindenfors et al. 2004). In this context, population surveys of howler monkeys in forest fragments with regard to group size and female numbers enables inferences about social structure dynamics and variation (Aguiar et al. 2009), and local ecological pressures.

Howler monkeys live in social groups whose size, sex ratio, and age composition can vary across species and populations, though these demographic traits tend to be more consistent within species than between them (Crockett and Eisenberg 1987; Ingberman et al. 2009; Neville et al. 1988). Besides adult sex ratios, their social organization is also influenced by risks of predation, infanticide, disease outbreaks, by group histories, limited dispersal in fragmented habitats, and human hunting (Chapman and Pavelka 2005; Crockett and Janson 2000; Knopff and Pavelka 2006; Pope 2000; Steenbeek and van Schaik 2001; Treves and Chapman 1996; Urbani 2005). Among some howler species, increased group sizes have been associated with habitat fragmentation, presumably due to constrained dispersal opportunities (Pope 2000). Conversely, reductions in group size and in the number of adults can occur in groups and populations during the early stages of establishment or while recovering from disease outbreaks (Clarke et al. 2002; Gorostiaga et al. 2025; Mendes 1989; Miranda et al. 2006; Ostro et al. 2001).

Howler societies allow for both female and male dispersal from their natal groups around sexual maturity, either to join established groups or form new, smaller ones (Ostro et al. 2001; Strier 2021). New groups typically form through one of two primary mechanisms: the fission of larger groups or the coalescence of dispersing individuals (Fedigan and Jack 2001; Treves 2001). Howler monkey groups can be constituted by just one or by multiple adult males, but generally with more females than males (Crockett and Eisenberg 1987), and their mating systems are predominantly polygynous or polygynandrous (Kowalewski 2007), often involving a dominant male attempting to monopolize mating opportunities with adult females in the group. However, extra-group copulations have been observed in both sexes (Decker and Bicca-Marques 2013). Interestingly, although the number of females tend to increase with the number of males, while group size increases (Crockett and Eisenberg 1987), male and female reproductive success can decrease with group size and with the number of adults of its respective sex in trans-Andean howler monkey species (*Alouatta palliatta* and *Alouatta pigra*; Treves 2001; van Belle and Estrada 2008). But male reproductive success can increase with the number of males in the group due to the increase of immature survival, probably resulting from more protection against infanticide in Amazonian red howler monkeys (*Aloutta seniculus*; Pope 1990).

The brown howler monkey (*Alouatta guariba*) is distributed throughout the Brazilian Atlantic Forest, from the northeast to the southernmost limits of its range, and westward to the northeastern Argentina (Oklander et al. 2024). The species typically occurs in fragmented landscapes across their range, including urban and peri-urban remnants, where they face a variety of anthropogenic threats (Chaves and Bicca-Marques 2013; Chaves et al. 2022; Oklander et al. 2022). Social groups range from two to 13 individuals, with a consistent mean of approximately five to six members across studied populations (Ingberman et al. 2009; Jardim 2005; Miranda and Passos 2005; Mendes 1989; Oklander et al. 2022), even when animals are in low abundance (Ferreguetti et al. 2020). Their social organization typically follows a one-male and multi-female structure, with adult sex ratios of one male to every two or three females, alongside varying numbers of juveniles and infants (Ferreguetti et al. 2020; Ingberman et al. 2009; Jardim 2005).

Brown howler monkeys were listed among the world’s 25 most endangered primate taxa during 2012 to 2023 (Mittermeier et al. 2022), due to extensive habitat loss and their extreme vulnerability to sylvatic yellow fever outbreaks (Bicca-Marques et al. 2017; Oklander et al. 2022). In Paraná state, southern Brazil, the species is classified as critically endangered (Paraná 2025). In Curitiba—the state capital—and its metropolitan region, they are found in small remnants of Araucaria Pine Forest (APF; Mixed Ombrophilous Forest; Sukow et al. 2025), a subtropical Atlantic Forest ecosystem that has been reduced to just 4% of its original extent and is nearing ecological extinction (Zorek et al. 2024), and where studies about social organization of howlers are rare (Miranda et al. 2006; Miranda and Passos 2005), limiting our ability to assess crucial populational informations to develop effective local conservation.

In this study, we investigated social organization—specifically group size and sex and age composition—of brown howler monkey groups living in three small nearby APF fragments in Curitiba, southern Brazil. To assess the groups’ status and social dynamics, we investigated whether: (1) the number of males, females, and immatures varies with group size; (2) the number of individuals of each sex are correlated with one another; and (3) the number of immatures is related to the number of adult females and males within the surveyed groups.

## Methods

### Ethical statement

This study was approved by the Curitiba Municipal Department of the Environment (SMMA Curitiba; no permission ID was issued) and had verbal permission of farm owners. Data collection adhered to all local ethical and legal guidelines, and to the Code of Best Practices for Field Primatology by International Primatological Society. We used AI to review the English language of the text.

### Study sites

We surveyed brown howler monkey groups in three relatively small (40–63 ha) peri-urban forest fragments of APF (Ombrophilous Mixed Forest) in the same landscape situated three to seven kilometers apart. The fragments are in the Municipal Protection Environmental Area of the Iguaçu River. This protected area lies in the southern region of Curitiba city, state of Paraná, southern Brazil.

Fragment 1 (25°33′35′′S, 49°13’53′W) is located within the Municipal Park of Iguaçu, which also serves as a buffer zone for the Municipal Zoo of Curitiba. On its western side, it borders a privately owned forested area belonging to the Bethânia Community, and together, these two areas comprise a 63-ha forested region, being the largest study fragment. Most of the forest is in a secondary successional stage, but also includes well-preserved patches dominated by dense stands of large, mature araucaria pines (*Araucaria angustifolia*), which contribute to a tall, continuous, emergent canopy. Because much of this forest is under the jurisdiction of a municipal park, it has historically been better conserved and subjected to less anthropogenic disturbance than the other study sites (described below). However, during fieldwork, we occasionally encountered signs of human activity—including makeshift camps, hunters, dogs and other evidence of illegal hunting.

Fragment 2 (25°34′55"S, 49°15′39"W) is a 53-ha privately owned forest surrounding a farm (Nazareno farm). The structure of this forest is less vertically complex, with fewer, smaller, and more widely spaced patches of araucaria pines. This forest is subject to more intense deforestation pressure due to adjacent urban development and agricultural activities, resulting in higher levels of human disturbance. Cattle and dogs are observed using the interior of the forest.

Fragment 3 (25°36’32"S 49°19’32"W) has 40 ha of forest and belongs to the Pinhal do Santana Municipal Park. It is the smallest and more elongated fragment with higher surface/edge ratio. This is a brand new park, recently established in 2022. It is open to the public from 8 am to 7 pm, and has interpretive trails for visitors as well as other unmarked trails within the forest. The park has some patches of mature araucaria pines and a small reforestation patch of exotic pinus, in which brown howlers can use (GR Santos Mariano, personal observation). Dogs and illegal baiting to hunting was frequently observed in the park. Finally, to the best of our knowledge, howler monkeys are not provisioned and do not rely on anthropogenic foods in any of the fragments.

### Data collection and analyses

Howler monkeys were surveyed through direct observation during weekly walks along established trail systems within the forest fragments and around their perimeters (Abreu et al. 2019), at approximately 1 km/h, aided by binoculars. We did not resample the trail system of the same fragment on the same field day.

Animals were also located by following the direction of their roars—a long-distance call that serves various ecological and social functions, including intergroup spacing, predator deterrence, rival and mate assessment, and ritualized displays during group encounters (Grasseto and Jalles-Filho 2007; Neville et al. 1988). This allowed the observers to find and to approach the vocalizing individuals for observations and counts.

We used a compass and GPS to ensure accurate spatial referencing and to record the locations of animal encounters. Surveys were conducted in the morning (8:00–12:00), afternoon (13:00–18:00), or throughout the day (8:00–17:00) when possible. In fragments 1 and 2, data were collected over 13 consecutive months, from September 2023 to September 2024. In fragment 3, data were collected during 11 consecutive months, from November 2024 to September 2025.

Each time we found the howlers, we spent as many times as possible with them to ensure the count and identify the individuals. Repeated encounters with identified individuals in a given area were mapped in Google Earth to delineate the spatial segregation between groups. We observed howlers found customarily in close proximity to one another in the same or in neighboring trees, isolated from other groups of individuals during the sampling day. So, we define a group following Kaplin and William (2013), as an aggregate of interacting individuals that exhibit cohesion within a given area, being closer to one another than to outsiders, and by engaging together in synchronized behaviors such as resting, moving, fleeing, and vocalizing during our observations.

Brown howler monkeys display marked sexual dimorphism in body mass and coat coloration, with adult males being significantly heavier and with vivid orange pelage. Females and immature individuals retain a darker brown coloration (Gregorin 2006; Neville et al. 1988; Oklander et al. 2024). So, animals were classified into sex and age categories based on morphological and behavioral characteristics, such as body size, coat coloration, and behavioral patterns. The categories used were adult males (AMs), subadult males (SMs), adult females (AFs), juveniles (Juvs), and infants (Infs). Individuals—predominantly males—and their respective groups were identified through analyses in photographs of their bodies and, especially, their facial characteristics, including facial contour, pelage coloration patterns, beard dimensions, head tuft morphology, lip and nostril shape, eyebrow prominence, and the presence or spatial distribution of scars.

Based on the similarity in composition among the groups we found and in fragment sizes, composition of observed groups in the three fragments were combined for analyses. The number of individuals in each group or sex-age classes were used to calculate mean group size and the proportional representation of each sex and age category. The immature-to-adult female ratio is a proxy of reproductive ratio and can reflect the accumulated survival rate of immatures in the groups (Treves 2001). It was estimated by calculating the ratio of Infs plus Juvs to the number of Afs (Ferreguetti et al. 2020; Jardim 2005; van Belle and Estrada 2008). Sex and age ratio was calculated dividing the number of individuals of each category by the number of adult males.

To test whether the number of individuals in each sex and age class varies with group size and between classes, we fitted five separate General Linear Mixed Models (GLMMs), with negative binomial distribution, using the fragments as random effects. First, to assess whether group size predicts the number of males (AMs + SMs) and AFs, and whether one of these classes predicts the another, we constructed two GLMMs. In the first model, group size and the number of males were used as predictor variables, with the number of AFs as the response variable. In the second model, group size and the number of AFs served as predictors, with the number of males as the response variable. Subsequently, to evaluate whether group size and the number of males and AFs predict the number of juveniles (Juvs), infants (Infs), and immatures (Juvs + Infs), we built three additional GLMMs. In each of these models, group size, the number of males, and the number of AFs were included as predictors, while the number of Juvs, Infs, and immatures were the response variables, respectively. We adopted the category of “males” as the total number of AMs and SMs in each group, since the numbers of these classes per group were small and showed little variation. All statistical analyses were performed in RStudio (R Core Team, 2024), with statistical significance set at *p* < 0.05.

## Results

We recorded 83 individuals distributed across 17 distinct social groups, and five apparently solitary individuals (two AMs, two AFs, and one Juv), based on 56 independent encounters along 332 km walked in 405 h of field effort. Brown howler monkeys were followed and directly observed for approximately 111 h and 30 min. Seven social groups (G1–G7; Table 1) were identified in fragment 1, based on 26 encounters during which 170 km were walked and a cumulative 172 h of field effort were logged. In fragment 2, six additional groups (G8–G13; Table 1) were observed across seven encounters, with 50 km walked and 50 h of field effort. In fragment 3, we identified four social groups (G14-G17; Table 1) across 23 encounters, totaling 112 km of walking and 183 h of field effort.

**Table 1:** Group size, sex and age composition, hours of observation and locations of 17 social groups of brown howler monkeys surveyed in three Araucaria Pine Forest fragments in Curitiba, Paraná, southern Brazil

Groups	Adult males	Subadult males	Adult Females	Juveniles	Infants	Group size	Time of observations (h)	Study sites
G1	1	0	2	1	0	4	03:48	Fragment 1
G2	1	0	2	1	1	5	04:00	Fragment 1
G3	1	1	2	0	0	4	05:40	Fragment 1
G4	1	0	3	2	0	6	14:40	Fragment 1
G5	1	1	3	1	0	6	12:20	Fragment 1
G6	1	1	3	2	1	8	00:30	Fragment 1
G7	1	1	0	1	0	3	00:30	Fragment 1
G8	1	1	2	1	1	6	08:16	Fragment 2
G9	1	0	2	2	1	6	02:45	Fragment 2
G10	1	0	1	2	0	4	04:19	Fragment 2
G11	1	0	1	2	1	5	10:55	Fragment 2
G12	1	1	2	4	0	8	01:30	Fragment 2
G13	1	1	1	1	0	4	00:30	Fragment 2
G14	1	1	1	1	0	4	16:25	Fragment 3
G15	1	1	2	0	1	5	06:31	Fragment 3
G16	1	0	1	0	0	2	07:12	Fragment 3
G17	1	0	1	0	1	3	11:42	Fragment 3
Total	17	9	29	21	7		111:30	

Group sizes ranged from two to eight individuals (mean 4.88 ± 1.65 SD). Around 53% of the groups had two males, being one AM and one SM. The remaining groups have only one AM. The number of AFs varied 0–3, the number of Juvs varied 0–4, and of Infs varied 0–1. The proportion of sex and age classes was 1 AM: 0.5 SM: 1.7 AF: 1.2 Juv: 0.4 Inf, and the immature-to-female ratio was 0.96.

Regarding the GLMM analyses (Table 2), we found support for group size as a positive predictor of the number of AFs, based on our first GLMM model. In contrast, the number of males did not predict the number of females. We found that neither group size nor the number of AFs predicted the number of males in the second model. Also, we found no support that the number of AFs predicted the numbers of Juvs, Infs and immatures in general, according to the last three models. However, we found support for group size as a positive predictor of the number of Juvs and the number of immatures, according to the third and fifth models. Finally, the number of Infs was not predicted by any of the variables tested.

**Table 2:** General Linear Mixed Models (GLMMs) with negative binomial distribution and the forest fragments as random effects for the numbers of males (adult males + subadult males), adult females, juveniles, infants and immatures (juveniles + infants) in relation to group sizes and the classes of sex and age

Models	Response variables	Predictive variables	Estimates	Std. Errors	Confidence intervals	Z values	*P*
1	Adult females	Intercept	− 0.3896	0.6274	− 1.91, 1.01	− 0.621	0.5346
		Group size	0.2274	0.1136	− 0.007, 0.47	2.002	0.0452
		Males	− 0.1628	0.3772	− 0.96, 0.63	− 0.432	0.66
2	Males	Intercept	0.41864	0.19564	− 1.07, 1.31	2.14	0.034
		Group size	0.161	0.28771	− 0.22, 0.41	0.56	0.5757
		Adult females	− 0.08755	0.29574	− 0.75, 0.54	− 0.296	0.7672
3	Juveniles	Intercept	− 0.9823	0.7858	− 2.75, 0.63	− 1.25	0.21127
		Group size	0.6169	0.193	0.22, 1.02	3.197	0.00139
		Adult females	− 0.613	0.3902	− 1.42, 0.15	− 1.571	0.11623
		Males	− 0.6616	0.48	− 1.7, 0.33	− 1.378	0.16816
4	Infants	Intercept	− 12.387	13.972	− 4.32, 1.53	− 0.887	0.375
		Group size	0.3807	0.3479	− 0.33, 1.10	1.094	0.274
		Adult females	− 0.2449	0.67	− 1.63, 1.16	0.365	0.715
		Males	− 0.7964	0.8427	− 2.64, 0.85	− 0.945	0.345
5	Immatures	Intercept	− 0.4865	0.6795	− 4.95, -0.23	− 0.716	0.47397
		Group size	0.56	0.1553	1.19, 4.94	3.607	0.00031
		Adult females	− 0.5216	0.3204	− 1.28, 0.10	− 1.628	0.10349
		Males	− 0.6942	0.3987	1.39, 0.22	− 1.741	0.08163

Data are from 17 social groups of brown howler monkeys surveyed in three Araucaria Pine Forest fragments in Curitiba, Paraná, southern Brazil

## Discussion

The mean group size, the predominance of one-adult male groups, and the proportion of AFs to AMs observed in the 17 brown howler monkey groups surveyed across three small APF fragments were consistent with values reported for the species throughout various Atlantic Forest phytophysiognomies in Paraná state and elsewhere. Specifically in Paraná state, these parameters are closely aligned with those documented in inland fragments (Aguiar et al. 2008), inland continuous forests (Miranda and Passos 2005), and the coastal continuous rainforest on islands (Ingberman et al. 2009). Although higher group sizes have been reported in studies from the northern range of the species (Mendes 1989), and at its southern distributional limit (Ingberman et al. 2009; Jardim 2005), our findings reinforced that some of the social organization traits, such as the adult sex ratio, the presence of a single adult male per group, and the average group size as remarkably conserved across brown howler populations, including those inhabiting relatively small APF fragments.

However, our observations indicated a possible low population based on our small encounter rate (1.7 encounters/ 10 km), where larger groups were infrequent, and with no group containing more than three AFs (Table 1). In fact, we observed one group with no AFs, seven groups with just one AF, and four groups consisting of a single male-female adult pair. The small group organization with few females suggests the presence of recently established groups (Miranda et al. 2006; Mendes 1989), or alternatively, could reflect the limited carrying capacity of these small APF fragments.

The variation in the immature-to-adult female ratio documented in our study falls within the range reported in previous research on brown howlers (Ferreguetti et al. 2020; Ingberman et al. 2009; Jardim 2005; Mendes 1989; Miranda and Passos 2005.). However, the 0.96 immature: adult female ratio indicates a stagnant or even barely decreasing population, with no growth, at least during the time we sampled. Although this metric can be subject to bias—such as the influence of infant mortality and female migration—which may limit its accuracy in expressing reproductive rates, it is nonetheless widely considered a proxy for reproductive rate in studies of the genus *Alouatta* (e.g. Neville et al. 1988; Treves 2001), serving as a valuable measure for comparative analyses. Therefore, our data indicated a currently small, established population of brown howler monkeys living in relatively small APF fragments.

We found that group size emerged as a significant positive predictor for the number of AFs, Juvs, and immatures overall. These findings indicate that, in line with Lindenfors et al. (2004), adult females could constitute the core of group growth in this population of howler monkeys. Furthermore, the larger groups appear to confer a fitness advantage, as evidenced by the positive relationship between group size and the number of immatures per group—again, a proxy for reproductive rate. It is possible that group size predicted the number of immatures due to the alleviation of infanticide, predation or competition against other groups, and the increased chance of finding food (Markham and Gesquiere 2017). At the same time, larger groups could be more attractive to females join them. On the other hand, no other independent variable tested predicted the number of males and the number of infants, possibly because of the small quantity and limited variation of their numbers in each group (Table 1)—and the ephemeral or short duration of the life stage of the latter age category.

In conclusion, our findings show that the size and composition of brown howler monkey groups across peri-urban fragments of APF are consistent with the social organization of the species, though small groups with less adult females were more frequently observed. Our findings confirm the presence of a currently small, established, but possibly stagnant, population of an endangered monkey species in an endangered Atlantic Forest ecosystem, within an Environmental Protection Area, where human activities should be more restricted. Although anthropogenic pressure continues to impact these forests, immediate action to control such activities and to connect this fragmented population of howlers is crucial to ensure the long-term survival of these primates and their equally threatened forest habitats.
